# Blastomere/trophectoderm biopsy for preimplantation genetic testing does not affect early HCG levels

**DOI:** 10.5935/1518-0557.20260029

**Published:** 2026

**Authors:** Heli Alexandroni, Eadit Buhbut, Yovel Cohen, Gheona Altarescu, Talia Eldar-Geva, Ido Ben-Ami

**Affiliations:** 1 IVF and Infertility Unit, Department of Obstetrics and Gynecology, Shaare Zedek Medical Center, Jerusalem, affiliated with the Faculty of Medicine, Hebrew University of Jerusalem, Israel. The Eisenberg R&D Authority, Shaare Zedek Medical Center; 2 Faculty of Medicine, Hebrew University of Jerusalem, Israel; 3 Medical Genetics Institute, Zohar PGD Unit, Shaare Zedek Medical Center, Jerusalem 9103102, Israel; Faculty of Medicine, Hebrew University, Jerusalem 9112102, Israel; 4 Infertility and Reproduction Department, Israeli Ministry of Health, Jerusalem, Israel (current position)

**Keywords:** HCG, PGT, blastomere biopsy, trophectoderm biopsy

## Abstract

**Objective:**

To evaluate if blastomere/trophectoderm biopsy for preimplantation genetic testing affects early HCG levels.

**Methods:**

A single-center retrospective study, encompassing data of 196 women who underwent IVF treatments with intracytoplasmic sperm injection (ICSI) with either a transfer of a fresh or frozen-thawed day-5 blastocyst. All women tested positive for HCG (>25 IU/L) two weeks after the transfer. Of those, 97 women underwent preimplantation genetic testing (“PGT”, the study group) while the control group (“non-PGT”) of 99 women, had IVF treatment without PGT.

**Results:**

No significant difference was found in HCG levels between the PGT and non-PGT groups. Similarly, there were no significant disparities in pregnancy outcomes, including miscarriage, live birth rate (LBR), extrauterine pregnancy, and intrauterine fetal death.

Multivariate analysis revealed that HCG levels were significantly associated with Body Mass Index (BMI) and protocol type (fresh vs. frozen), but not with the biopsy day. Logistic regression analysis adjusted to the women’s age, endometrial thickness, and HCG levels, found no significant correlation between the likelihood of live birth and PGT performance or the biopsy day.

**Conclusions:**

There is no apparent disparity in early HCG levels or pregnancy outcomes in IVF treatment with or without PGT. Notably, HCG levels are significantly higher in frozen-thawed cycles compared to fresh cycles, irrespective of PGT performed. Since HCG levels are associated with LBR, these findings enhance confidence of the non-detrimental effect of blastomere and trophectoderm biopsy.

## INTRODUCTION

Biopsy for preimplantation genetic testing (PGT) has become foundational in assisted reproductive technology, enabling the screening and identification of genetic abnormalities in embryos before embryo transfer (ET). Since the advent of chromosomal and molecular analysis in the early 1990’s (Handyside *et al*., 1990; Munné *et al*., 1993), PGT has evolved significantly, becoming a standard procedure for couples at high genetic risk of transmitting genetic disorders or seeking to exclude embryos with chromosomal aberrations. PGT involves the biopsying of polar bodies, blastomeres at the cleavage stage (day 3), or trophectoderm cells at the blastocyst stage (day 5-6) of embryo development. Among the possibilities, trophectoderm biopsy is the main procedure performed today.

As syncytiotrophoblast cells, essential for human chorionic gonadotropin (HCG) secretion, originate from trophectodermal fusion, concerns have emerged regarding the potential impact of biopsy procedures on initial HCG levels and subsequent pregnancy outcomes.

HCG is a glycoprotein hormone that plays a critical role in implantation and early trophoblast decidual invasion (Makrigiannakis *et al*., 2017). Its functions include promoting progesterone production by corpus luteal cells and supporting the development of the placenta, uterus and fetus. Sensitive reverse transcription-polymerase chain reaction (RT-PCR) techniques allow for HCG transcription detection as early as the two-cell stage embryo, with increased expression noted in the blastocyst stage (Jurisicova *et al*., 1999). Previous studies have examined the predictive value of early serum HCG levels in assessing pregnancy outcomes (Homan *et al*., 2000; Lawler *et al*., 2011). Most of these studies indicated a positive correlation between HCG levels and ongoing pregnancy rates in both natural cycles (Barnhart *et al*., 2004; Sasaki *et al*., 2008) and following in vitro fertilization (IVF) treatments (Wang *et al*., 2020). The relationship between various aspects of IVF treatments and HCG levels has been investigated before; for example, pregnancies attained through intracytoplasmic sperm injection (ICSI) have been associated with lower HCG levels compared to those achieved through conventional IVF, particularly in couples with unexplained infertility (Gold *et al*., 2000; Poikkeus *et al*., 2002).

Conflicting findings have emerged regarding HCG levels following fresh versus frozen-thawed ET (Poikkeus *et al*., 2002; Reljič *et al*., 2013; Morse *et al*., 2016; Grin *et al*., 2019). Proposed hypotheses include a potential negative impact of embryo cryopreservation and thawing on trophoblast integrity in frozen cycles that could explain lower HCG levels in frozen cycles (Reljič *et al*., 2013), and the adverse effect of a non-physiologic maternal environment on trophoblast differentiation and placentation in fresh cycles that could explain lower HCG levels in fresh cycles (Morse *et al*., 2016). Despite these insights, the establishment of specific thresholds for HCG levels in early pregnancies as a predictor of clinical pregnancy, ongoing pregnancy, or live birth remains an area for further investigation, influenced by factors such as the timing of HCG measurement and the age of the transferred embryo (Oron *et al*., 2015; Kathiresan *et al*., 2011). In assessing early HCG levels, studies often reference measurements taken between 12 to 16 days after ET (Lawler *et al*., 2011; Wang *et al*., 2020; Gold *et al*., 2000; Poikkeus *et al*., 2002; Reljič *et al*., 2013) or 15-16 days following ovum retrieval or ovulation (Homan *et al*., 2000; Morse *et al*., 2016).

Given the psychological burden of fertility treatments, alongside the importance of early measurements of HCG levels as a predictor of pregnancy outcomes, it is essential to investigate the relationship between biopsy procedures and early HCG levels.

We aim to compare early HCG levels following blastocyst transfer among women undergoing IVF treatments with and without blastomere or trophectoderm biopsies, while also considering differences between fresh and frozen IVF cycles.

## MATERIAL AND METHODS

### Study design

This retrospective case-control descriptive study was conducted in a tertiary IVF clinic, between 2020 and 2023. Data were gathered from patient’s medical records at the IVF unit and were encoded. The research was approved by the Institutional Ethics Committee (0257-22-SZMC) and adhered strictly to the principles outlined in the Declaration of Helsinki.

### Study participants

The study population included women who tested positive for HCG (>25 IU/L) two weeks after a single blastocyst transfer. The study group comprised women who underwent either blastomere or trophectoderm biopsy (the “PGT group”), while the control group consisted of women who underwent IVF treatments without PGT (the “non-PGT group”).

Sample size estimation was based on the expected difference in HCG levels on day 14 between the PGT and non-PGT groups. Assuming that the minimally clinically significant difference between the groups is 400, with a Standard Deviation (SD) of 1230 in the PGT and 967 in the non-PGT groups (based on a previously published study (Durga *et al*., 2021)), a significance level of 5% (one-sided), and a power of 80%, a sample size of 100 women in each group was found to sufficient to prove that the difference between the groups is statistically significant. The choice to use a one-sided test is in accordance with previous studies that reported lower hCG levels following PGT (Cho *et al*., 2011, Lu *et al*., 2020). Since we hypothesized that PGT would be associated with lower, but not higher, hCG levels, a one-sided test was considered appropriate.

Inclusion criteria encompassed women aged 18-45 with a body mass index (BMI) of 18-35, who underwent fresh or frozen-thawed IVF protocols, fertilization via ICSI, and had a single blastocyst transfer. Exclusion criteria included egg donation, uterine malformation, HCG <25 IU/L, or transfer of more than one blastocyst.

Comprehensive medical histories were obtained from all available records, covering general and gynecological aspects, including the ages of the women and their partners, BMI, gravidity, parity, type of infertility (primary or secondary), infertility cause, PGT type, and the number of mutations necessitating PGT. Cycle-related variables included protocol type (fresh agonist, fresh antagonist, frozen-thawed natural or hormone replacement therapy (HRT)), the peak estradiol (E2) level and maximal endometrial thickness before blastocyst transfer, E2, and progesterone levels on HCG measurement day, and the presence of moderate to severe ovarian hyperstimulation syndrome (OHSS). For fresh blastocyst transfer cycles, additional data regarding the gonadotrophin (GT) types and total dose administrated, number of follicles >14mm in ultrasound (US) prior oocyte pick-up (OPU), number of aspirated mature oocytes (metaphase II, M2), number of fertilized eggs (two pronuclei, 2PN), and fertilization rate were also assessed.

### IVF protocols and embryo biopsy

Controlled ovarian stimulation was performed using Gonadotropin-releasing hormone (GnRH) agonist or antagonist cycles. The protocol choice was individualized based on the patient’s characteristics, such as age, ovarian reserve, and previous cycle history, as determined by healthcare providers. Oocyte retrieval was performed under transvaginal US guidance, followed by ICSI. Embryos were cultured in a single-step medium (“Global total”) at 37°C with 5% CO_2_ and 5% O_2_. Blastocyst quality was graded as “good,” “fair,” or “poor,” defined by the Gardner scoring system.

In cases requiring PGT, biopsy of cells was performed at the blastomere or blastocyst stages, using 1000-µs laser pulses (Lykos; Hamilton Thorne) followed by cell aspiration. When biopsies for PGT were performed on day 3, a single blastomere was removed, and the cells were sent for genetic analysis using the polymerase chain reaction (PCR) method.

The embryos were then cultured to the blastocyst stage, by which time the genetic results were available, allowing the transfer of fresh blastocysts or their cryopreservation by vitrification for future frozen-thawed cycles. When biopsies were performed on day 5, five to seven trophectoderm cells were removed from the blastocysts for genetic analysis using either the PCR or Next-Generation Sequencing (NGS), followed by cryopreservation of the biopsied blastocysts.

Thawed blastocysts were transferred in either HRT cycles, treated with oral E2 and vaginal progesterone, or in natural cycles. The protocol choice was individualized, based on the patient’s characteristics, as determined by healthcare providers. All blastocyst transfers were performed under US guidance, and HCG testing was determined 13-15 days post-blastocyst transfer.

### Outcomes

The primary outcome measured was HCG levels 13-15 days after blastocyst transfer. Secondary outcomes encompassed various parameters such as rates of clinical pregnancy (confirmed by the presence of a gestational sac in the US), chemical pregnancy (HCG >25 IU/L without a gestational sac), miscarriage (defined as a non-viable fetus before 24 weeks), extrauterine pregnancy (EUP), intrauterine fetal demise (IUFD, defined as stillbirth after 22 weeks of pregnancy), and live birth (defined as birth occurring after 24 weeks). These outcomes were analyzed within the two study groups.

### Statistical analysis

A dependent T-test was employed to compare quantitative data between the PGT and non-PGT groups. The relationship between categorical variables was determined using the Chi-square Test or Fisher’s exact test. The Mann-Whitney Test was utilized to evaluate the peak E2 levels, as well as E2 and progesterone levels at HCG measurement day and miscarriage time.

Correlation analysis between HCG levels and quantitative variables was conducted using the Pearson correlation coefficient. Categorical variables correlations were assessed using an independent T-test. Additionally, a two-way analysis of covariance was performed for HCG levels using a multivariate model.

To compare the route of progesterone administration for hormonal support and HCG levels, a non-parametric ANOVA Kruskal-Wallis test was applied.

Statistical calculations excluded missing data and a significance level of *p*-value<0.05 was considered statistically significant. The statistical analyses were conducted using the “IBM SPSS Statistics, version 27” (2020) software.

## RESULTS

A total of 196 women were included in the study, 97 (49.5%) of them were in the PGT group and 99 (50.5%) in the non-PGT group. The baseline characteristics of the women are detailed in Supplementary Table 1.

There were no significant differences in age and BMI in the two groups. However, gravity and parity rates were notably higher among women in the PGT group compared to the non-PGT group (2.1 *vs*. 1 for gravity, and 1.5 *vs*. 0.7 for parity, *p*<0.001).

Cycle characteristics are presented in Table 1. Women in the PGT group received higher total doses of GT compared to those in the non-PGT group (2338±1163 units *vs.* 1797±697, *p*=0.018), and had more oocytes retrieved (16.5±8.2 *vs*. 11.6±4.2, *p*=0.006). In addition, the PGT group had statistically higher numbers of M2 oocytes and fertilized eggs (2PN) compared to the non-PGT group (13.7 *vs*. 10 and 10.8±6.0 *vs*. 6.8±3.5, *p*<0.01). The fertilization rate was also significantly higher in the PGT group (77.4±16.7% *vs*. 66.8±21.7%, *p*<0.05). Notably, there was a significant difference in blastocyst grading with most blastocysts in the PGT group graded as “Good,” while those in the non-PGT group were predominantly “Fair” (*p*=0.033). No differences were observed in the type of GT administered, the incidence of OHSS, or hormonal support between the groups.

**Table 1:** Cycle characteristics of IVF treatments in the study groups

	Non-PGT (N=99)	PGT (N=97)	*p*-value
GT ingredient FSH FSH+LH HP HMG	22/26 (84.6%) 1/26 (3.8%) 3/26 (11.5%)	20/30 (71.4%) 4/30 (14.3%) 4/30 (14.3%)	NS
GT dose (units)	1797.63±697.08, 1795 (700-3750)	2338.8±1163.3, 2100 (900-6750)	0.04
Max. endometrial thickness (mm)	10.1±1.8, 9.8 (5.6-15)	9.8±2.1, 9.6 (5.7-15)	NS
Number of follicles >14 mm	10.8±4, 10 (5-23)	11.97±5.6, 11 (2-26)	NS
Number of retrieved oocytes	11.5±4.2, 11 (5-19)	16.5±8.2, 14 (1-41)	<0.01
M2	10±3.7, 9 (4-17)	13.6±7.2, 13 (0-36)	0.01
2PN	6.81±3.5, 6 (1-16)	10.8±6, 10.5 (0-29)	<0.01
Fertilization rate (%)	66.8±21.7, 71.4 (25-100)	77.4±16.7, 80.5 (25-100)	0.04
E2 max before OPU (pmol\L)	2493.2±3057, 938 (256-11643)	3353.7±3913.6, 1158 (125-15944)	NS
E2 on HCG day (pmol\L)	2878.6±3372.2, 1215 (126-18350)	2144.8±2031, 1427 (7-9784)	NS
P on HCG day (nmol\L)	66.5±41.3, 48 (2.3->127)	67.6±42.3, 54.7 (1.5-190)	NS
Blastocyst grade Good Fair Poor	42/82 (51.2%) 39/82 (47.2%) 1/82 (1.2%)	60/93 (64.5%) 28/93 (30.1%) 5/93 (5.3%)	0.03
Mild OHSS	0	4/97 (4.1%)	NS
Hormonal support PV progesterone PO progesterone IM progesterone Mixed progesterone Other	54 (54.5%) 5 (5.1%) 9 (9.1%) 4 (4%) 22 (22.2%)	60 (61.9%) 10 (10.3%) 9 (9.3%) 5 (5.2%) 10 (10.3%)	NS

Note: Mean±SD, median (range) is shown for continuous variables, and percentages are shown for dichotomous variables.

E2 = estradiol, FSH = follicle stimulating hormone, GT =gonadotropin, HCG = human chorionic gonadotropin, HP HMG = highly purified human menopausal gonadotropin, IM = intramuscular, LH = luteinizing hormone, M2 = metaphase 2, NS = not significant, OHSS = ovarian hyperstimulation syndrome, OPU = ovum pick-up, P = progesterone, PN = pronuclei, PO = oral, PV = vaginal.

### Primary outcome

HCG measurements were taken 14 days after blastocyst transfer from 155 women (79%, of which 75 in the PGT and 80 in the non-PGT groups), 13 days after blastocyst transfer from 37 (19%, of which 20 in the PGT and 17 in the non-PGT groups) women, and 15 days after blastocyst transfer from four women (2%, two women in each group). Comparison of median HCG values across these measurement days between the PGT and non-PGT groups revealed no significant differences, allowing for the inclusion of all values in statistical analyses.

**Figure 1:**
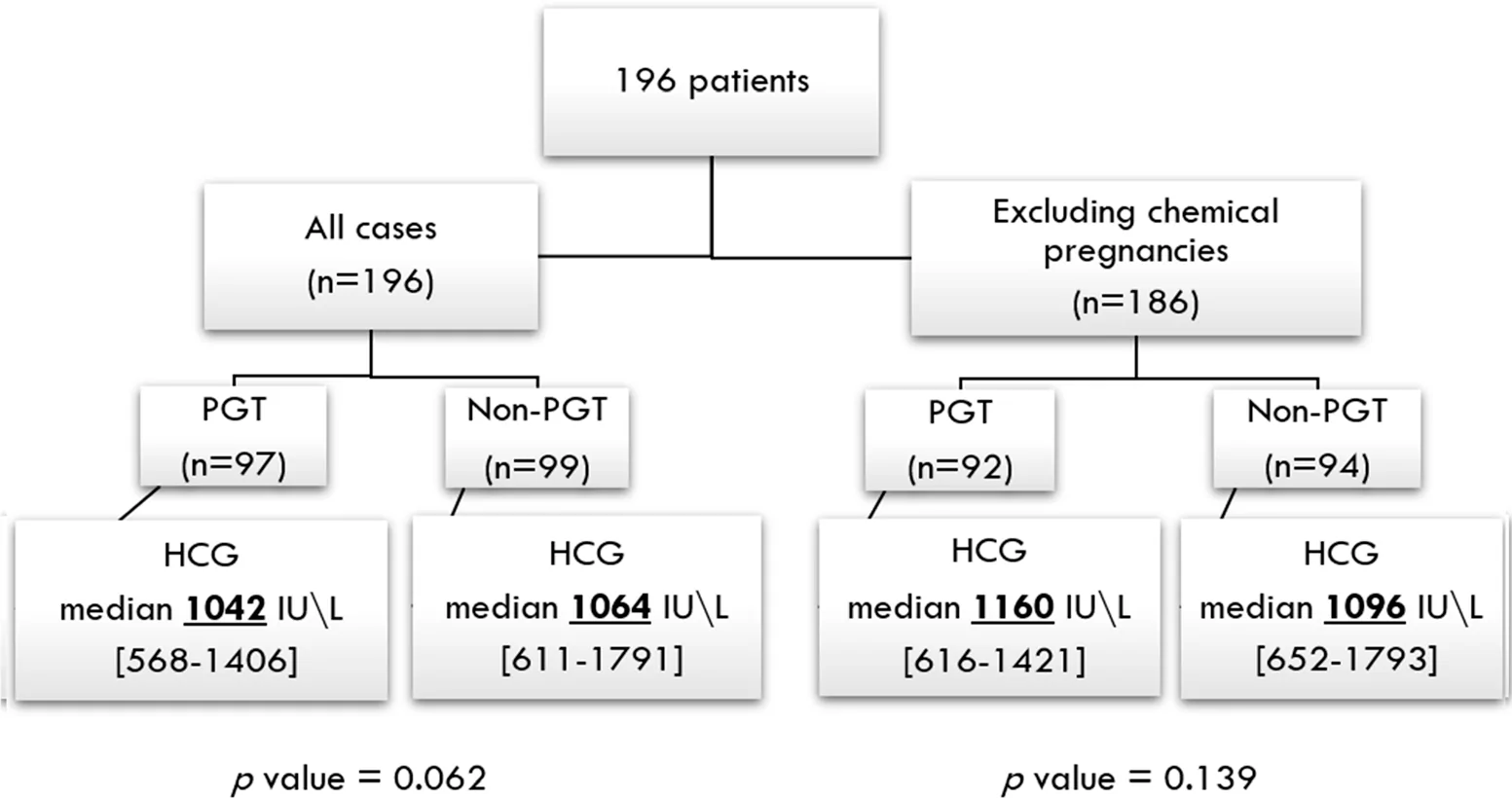
The median HCG for the PGT group and for the non-PGT group. No statistically significant difference between the groups was found.

The median HCG was 1042 IU/L for the PGT group and 1064 IU/L for the non-PGT group as illustrated in Figure 1, and no statistically significant difference between the groups was found. Ten cases of chemical pregnancies were identified, with nine exhibiting extremely low HCG levels (<100IU/L). To ensure accuracy, HCG levels were recalculated excluding these cases, resulting in similar medians (1160 *vs*. 1096, *p*=0.139).

### Comparison of HCG levels based on IVF protocol

After excluding chemical pregnancies from the statistical calculations, 56 women underwent fresh blastocyst transfer (“Fresh cycle”) and 130 women underwent frozen-thawed blastocyst transfer (“Frozen-thawed cycle”), as detailed in Table 2.

**Table 2:** HCG levels based on IVF protocols and biopsy day

Type of cycle	IVF protocol	PGT status and day of biopsy	Number of -patients	HCG levels (IU/L) Median (IQR)	*p*-value
Fresh cycles	Both protocols (agonist+antagonist)	Non-PGT	27	787 (557-1045)	NS
		PGT	29	1012 (621-1362)	
	Fresh agonist	Non-PGT	2	1019 (895-1144)	NS
		PGT	10	1141 (760-1589)	
		3	10	1141 (760-1589)	
		5	0		
	Fresh antagonist	Non-PGT	25	761 (557-1043)	NS
		PGT	19	860 (539-1218)	
		3	19	860 (539-1218)	
		5	0		
Frozen-thawed cycles	Both protocols (natural+medicated)	Non-PGT	67	1542 (710-1953)	0.028
		PGT	63	1077 (621-1425)	
	Frozen-thawed natural	Non-PGT	37	1577 (714-1939)	NS
		PGT	32	1226 (734-1530)	
		3	20	1047 (817-1404)	NS
		5	12	1333 (650-1747)	
	Frozen-thawed HRT	Non-PGT	30	1271 (746-1959)	NS
		PGT	31	1023 (451-1390)	
		3	13	1077(410-1375)	NS
		5	18	863 (495-1335)	
Fresh cycle Frozen-thawed cycles	All protocols	All cases (PGT+Non PGT)	56 13	(561-1154) 1203 (656-1793)	<0.01

HCG = human chorionic gonadotropin, HRT = hormone replacement therapy, NS = not significant.

**Figure 2:**
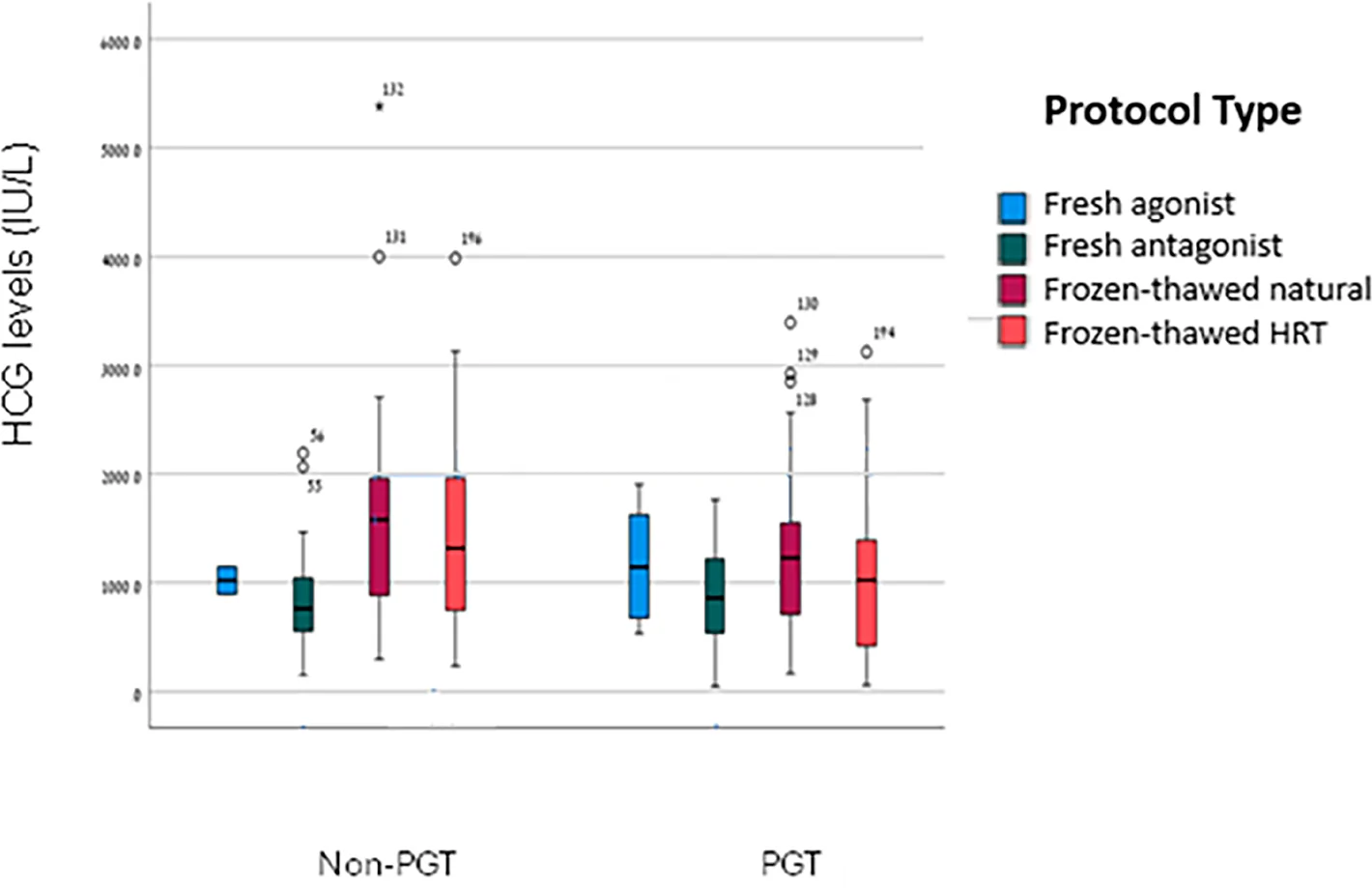
The comparison between HCG levels across different IVF protocols.

Figure 2 illustrates the comparison between HCG levels across different IVF protocols. When comparing the PGT and non-PGT groups within each specific protocol (fresh agonist, fresh antagonist, frozen-thawed natural, and frozen-thawed HRT), no significant differences were observed between the groups.

In fresh cycles, no significant difference in HCG levels was found between the PGT and non-PGT groups, even when analyzed by agonist or antagonist protocol. In frozen-thawed cycles, the median HCG levels were higher in the non-PGT group compared with the PGT group (*p*=0.028). There were no significant differences in HCG levels when analyzed by frozen-thawed natural or HRT protocols or by biopsy day. In addition, when comparing frozen-thawed and fresh blastocyst transfers, regardless of PGT status, higher HCG levels were found in the frozen-thawed cycles (*p*<0.01).

### Secondary outcomes

#### Pregnancy outcomes

There were no significant differences in the pregnancy outcomes between the PGT and non-PGT groups regarding live births, miscarriages, IUFD, EUP, and chemical pregnancies, as shown in Table 3. Additionally, the rates of vaginal and cesarean deliveries, as well as average birth weights, did not vary significantly between the study groups. Of the women included in the study, 146 (74%) had live births, with 71 (73%) in the PGT group and 75 (75%) in the non-PGT group. There was no significant difference in LBR between the PGT and non-PGT groups based on the protocol type (fresh *vs*. frozen-thawed). The analysis revealed no significant differences in pregnancy outcomes based on the presence of primary or secondary infertility between the PGT and non-PGT groups. Five cases of IUFD were recorded: two (2%) in the PGT group and three (3%) in the non-PGT group.

**Table 3:** Secondary outcomes: A. Obstetric outcomes in the PGT and non-PGT groups, B. Live birth rate based on PGT characteristics.

A. Obstetric outcomes			
	PGT (n=97)	Non-PGT (n=99)	*p*-value
Pregnancy outcome Clinical pregnancy Chemical pregnancy Extrauterine pregnancy	92/97 (95%) 5/97 (5%) 0 (0)	94/99 (95%) 5/99 (5%) 3/99 (3%)	NS NS NS
IUFD	2/97 (2%)	3/99 (3%)	NS
Miscarriage	19/97 (19%)	13/99 (13%)	NS
Live birth All cycle Fresh cycles Frozen-thawed cycles	71/97 (73%) 24/29 (82%) 47/68 (69%)	75/99 (75%) 23/27 (85%) 52/72 (72%)	NS
Mode of delivery Vaginal delivery Cesarean section	60/72 (83.4%) 12/72 (16.6%)	60/76 (78.9) 16/76 (21.1)	NS
Gestational age at delivery (weeks)	39±1	38+6±3.0	NS
Birth weight (grams)	3199.5±556.5	3204.6±527	NS

B. Live birth in the PGT group		
	Live birth (n=72)	*p*-value
Biopsy day Day 3 Day 5	48/63 (76.1%) 24/34 (70.5%)	NS
Type of PGT PGT-A PGT-M PGT A+M	13/18 (72.2%) 54/74 (72.9%) 4/5 (80%)	NS
Mutation carrier Maternal Paternal Both	25/36(69.4%) 20/27 (74.1%) 6/9 (66.7%)	NS
Number of mutations 1 2 3	66/87 (75.9%) 5/9 (55.6%) 0/1	NS
Infertility type Primary infertility Secondary infertility	4/8 (50%) 1/4 (25%)	NS
Blastocyst grade Good Fair Poor	45/60 (75%) 21/30 (70%) 3/6 (50%)	NS

IUFD = intrauterine fetal death, NS = not significant, PGT-A = preimplantation genetic testing for aneuploidy, PGT-M = preimplantation genetic testing for monogenic disorders.

IUFD = intrauterine fetal death, NS = not significant, PGT-A = preimplantation genetic testing for aneuploidy, PGT-M = preimplantation genetic testing for monogenic disorders.

#### Effect of PGT characteristics on live birth rate (LBR)

Characteristics of PGT cycles are summarized in Table 3. In the PGT group, considering the limited sample size, no statistically significant associations were found between LBR and biopsy day, presence of additional primary or secondary infertility, the number of mutations necessitating PGT, mutation carrier parents, and PGT type -whether for monogenic disorders (PGT-M), aneuploidy (PGT-A), or both.

#### Correlation between HCG levels and other variables

A Pearson correlation coefficient was calculated to assess relationship between HCG levels and various variables including women and their partners’ ages, BMI, gravity, parity, duration of infertility, number of follicles >14 mm, number of retrieved oocytes, number of M2 oocytes and 2PN fertilized eggs, maximal E2 levels before OPU, endometrial thickness before blastocyst transfer, and levels of E2 and progesterone on the day of HCG measurement. A significant negative correlation was identified between HCG levels and BMI (correlation coefficient of -0.226, *p*<0.001) and between HCG levels and maximal E2 values before OPU (correlation coefficient of -0.195, *p*=0.014).

HCG levels were not significantly influenced by the history of abortions, infertility diagnosis, progesterone support (including its route of administration), estrogen support, or timing of HCG measurement (Day 13 or 14). Additionally, women’s age did not impact HCG levels even when analyzed in subgroups of under and over 35 years.

A two-way analysis of covariance was performed on HCG levels using a multivariate model. Independent variables included those associated with HCG levels in univariate analysis, plus an additional clinically relevant variable (biopsy on day 3 vs. day 5). Results indicated that HCG levels were significantly influenced by the IVF protocol (frozen-thawed or fresh, *p*=0.03) and BMI (*p*=0.004), but not by the performance of PGT, biopsy day 3 *vs*. 5 or maximal E2 levels before OPU.

#### Association between live birth and other variables

A univariate analysis identified four variables significantly associated with live birth rates. First, maternal age emerged as a significant predictor for LBR, with a higher proportion of women under 35 achieving live birth compared to those aged 35 and above (76% *vs*. 57%, *p*<0.05). Second, increased endometrial thickness before blastocyst transfer correlated positively with live birth rates (10.1±1.9 mm *vs*. 9.4±2.1 mm, *p*<0.05). Third, higher progesterone levels on the day of HCG measurement were linked to better LBR (71.74±42.16 *vs*. 50.69±35.15 nmol/L, *p*=0.003). Finally, HCG levels were approximately twice as high in women who achieved live births compared to women with other obstetric outcomes - miscarriage, IUFD, or chemical pregnancy (1336.9±829.3 U/L *vs*. 792.89±660.26 IU/L respectively, *p*>0.01).

Subsequent to the univariate analysis, a logistic regression was performed to assess live births. The model incorporated the four significant variables mentioned above plus a fifth variable - PGT *vs*. non-PGT and biopsy day (3 *vs*. 5). While no significant difference was noted in LBRs for each additional year of maternal age, each millimeter increase in endometrial thickness was associated with an increased odds of live birth (Odds Ratio (OR) 1.289, Confidence Interval (CI) 1.037-1.601, *p*=0.022). Similarly, for every unit increase in HCG, there was a corresponding rise in the odds of live birth (OR 1.001, CI 1.001-1.002, *p*<0.01). In the second analysis, which included PGT status and biopsy day, we found that these factors did not significantly influence LBR when controlling for maternal age, endometrial thickness, and HCG levels.

## DISCUSSION

This study aimed to compare early HCG levels after blastomere or trophectoderm biopsies with non-PGT cycles, taking into account specific IVF parameters. Our findings indicate no significant differences in HCG levels between PGT and non-PGT groups.

Previous literature examining the relationship between biopsies for PGT and HCG levels has produced inconclusive results, pointing to various potential confounders.First, the expectation that HCG levels would be lower after a biopsy, due to the removal of cells capable of secreting HCG, depends on the timing of the measurement of HCG. For example, reduced HCG levels were measured on the 12th day after ET following blastomere (Cho *et al*., 2011) or trophectoderm biopsy (Lu *et al*., 2020) compared with pregnancies without PGT. However, Wu *et al*. utilized propensity score matching to equalize baseline characteristics and found that trophectoderm biopsy did not significantly affect serum HCG levels 14 days after ET (Lu *et al*., 2020). Moreover, Dokras *et al*. suggested that the removal of fewer than ten cells during trophectoderm biopsy does not significantly alter cumulative HCG secretion when assessed 14 days later (Dokras *et al*., 1991). This suggests that while early HCG levels may show a reduction shortly after biopsy, by 14 days post-transfer, any initial loss of cells is likely compensated, resulting in similar HCG levels between the PGT and non-PGT groups.

The type of IVF protocol may also influence HCG levels. Our study revealed that HCG levels were significantly higher in frozen-thawed cycles compared to fresh cycles (*p*<0.01) irrespective of PGT performed. This finding aligns with previous research indicating that a more stable and controlled hormonal environment in frozen-thawed cycles can enhance endometrial receptivity, facilitating early trophoblast differentiation and placentation (Reljič *et al*., 2013; Morse *et al*., 2016; Grin *et al*., 2019; Shapiro *et al*., 2011). Our finding of higher early HCG levels in frozen-thawed cycles in the non-PGT group compared to the PGT group may imply that the compensation timeline of HCG levels in frozen-thawed cycles takes longer than in fresh cycles. Another possible explanation is that, unlike in fresh cycles, the frozen-thawed cohort included transfers of blastocysts that had undergone day-5 trophectoderm biopsy. Lu *et al*. (2020) similarly reported lower HCG levels after blastocyst transfers in frozen cycles in the PGT group compared with non-PGT, attributing this to reduced HCG secretion following the removal of trophectoderm cells. In our sub-analysis comparing day-3 and day-5 biopsies of frozen cycles, we found that HCG levels were lower after day-3 biopsies in natural frozen-thawed cycles, whereas in HRT frozen-thawed cycles, HCG levels were lower after day-5 biopsies. Therefore, it cannot be concluded that HCG levels are universally lower following trophectoderm biopsy. Although the difference in HCG levels was statistically significant, the similar LBR in frozen-thawed cycles between the PGT and non-PGT groups. consistent with Lu et al.’s findings, indicates that this difference is clinically insignificant (Lu *et al*. 2020).

Additionally, HCG levels could also be affected by blastocyst grading. A recent publication by Li *et al*. (2022) stratified blastocysts based on trophectoderm morphological scores and assessed HCG levels 12 days after blastocyst transfer. They found that blastocysts with higher trophectoderm grades produced higher HCG levels, while those with lower trophectoderm grades showed a negative impact on HCG levels due to biopsy. (Li *et al*., 2022). In our study, although there were more “good” grading blastocysts in the PGT group compared to the non-PGT group (64.5% *vs*. 51.2%, *p*=0.033), there was no significant difference in HCG levels between the groups.

Furthermore, we found that HCG levels negatively correlated with maximal E2 levels before OPU and BMI. Notably, previous studies did not identify BMI and E2 as potential confounders for early HCG levels. This inverse relationship suggests that elevated E2 levels may create a hormonal environment that is less conducive to embryo implantation (Kalem *et al*., 2017), while a higher BMI might alter hormone distribution in adipose tissue or affect metabolic clearance rate, potentially impacting early pregnancy development (Wu *et al*., 2021).

The secondary outcomes of our study focused on pregnancy outcomes following IVF treatments. Regardless of HCG levels, we compared pregnancy outcomes between the PGT and non-PGT groups and found no significant differences in outcomes such as LBR, miscarriage, IUFD, or chemical pregnancy. This suggests that biopsy for PGT does not adversely affect obstetric outcomes, consistent with findings from previous studies (Li *et al*., 2022; Labarta *et al*., 2017; Hou *et al*., 2019; Özdamar *et al*., 2023). Furthermore, there were no significant differences in gestational age or birth weight between the two groups, corroborating earlier data (Eldar-Geva *et al*., 2014).

Previous research has indicated that higher HCG levels are associated with better obstetric outcomes, indicating that elevated HCG might reflect higher embryo quality, support proper placental development, signify successful implantation, and suggest a well-regulated endocrine environment, all of which enhance the chances of a successful pregnancy (Barnhart *et al*., 2004; Sasaki *et al*., 2008; Wang *et al*., 2020; Gold *et al*., 2000; Poikkeus *et al*., 2002; Oron *et al*., 2015; Kathiresan *et al*., 2011). Our logistic regression analysis aligns with these findings, validating HCG as a biomarker for predicting successful pregnancy outcomes. Specifically, each unit increase in HCG levels was associated with a higher probability of live birth, enhancing its predictive value.

It is important to note that LBR was also correlated with maternal age, endometrial thickness before blastocyst transfer and progesterone levels on HCG measurement day. Consistent with previous studies, higher progesterone levels correlated with improved endometrial receptivity and endometrial thickness, leading to better obstetric outcomes, including increased LBR (Labarta *et al*., 2017).

### Strength of the study:

The scientific validity and significance of our findings is strengthened by reviewing established relationships in the literature and employing advanced statistical analyses. Our methodology, which considers various aspects of IVF (such as biopsy day, IVF protocol and indication for PGT), provides a deeper understanding of how these aspects might affect pregnancy outcomes.

### Limitations of the study:

The retrospective design limits our ability to control all potential confounding factors that could influence HCG levels and pregnancy outcomes. Additionally, a limited sample size may reduce the statistical power of subgroup analyses. Another limitation is the inherent bias in patient selection and treatment allocation.

There were differences in baseline characteristics between the PGT and non-PGT groups, manifested as higher gravidity and parity and a lower incidence of infertility (consequently resulting in a better ovarian response and blastocyst quality) in the PGT group, reflecting a selection bias. These differences should be taken into account when interpreting the findings, even after multivariate adjustment.

## CONCLUSION

Our study strengthens confidence in the non-detrimental effects of cell biopsies for PGT. We demonstrate that these procedures do not negatively impact HCG levels or pregnancy outcomes and underscore the significance of HCG as a valuable prognostic biomarker for successful pregnancies and LBR. Future prospective studies should include larger cohorts, consider earlier HCG level measurements, and explore the long-term effects of PGT on pregnancy and child health.

## Figures and Tables

**Supplementary Table 1:** Baseline characteristics of study participants

	Non-PGT (N=99)	PGT (N=97)	*p*-value
Demographic Woman’s age (years) Partner’s age (years) BMI	30.5±4.7, 30 (23-42) 32.7±4.9, 33 (24-44) 24.6±4.3, 23.7 (19-34.2)	29.5±4.6, 30 (21-42) 31.5±5.2, 32 (21-53) 24.3±4.3, 23.6 (19-35)	NS NS NS
Obstetric history Nulliparous Parous Previous abortions >1	39 (40%) 60 (60%) 20 (20%)	19 (20%) 78 (80%) 24 (24.7%)	NS
Any Infertility Primary Secondary	84/99 (84.8%) 15/99 (15.2%)	8/97 (8.2%) 3/97 (3.1%)	<0.01
Infertility diagnosis Mechanical Male factor Unexplained Anovulatory Age-related Combined infertility	3/97 (3.1%) 52/97 (53.6%) 19/97 (19.6 %) 5/97 (5.2%) 3/97 (3.1%) 15/97 (15.5%)	0/11 4/11 (36.4%) 5/11 (45.5%) 0/11 0/11 2/11 (18.2%)	NS

Note: Mean±SD, median (range) is shown for continuous variables, and percentages are shown for dichotomous variables.

BMI = body mass index, NS = not significant.
